# Time efficiency, geometric accuracy, and clinical impact of AI-assisted contouring of organs at risk in head and neck cancer radiotherapy

**DOI:** 10.2340/1651-226X.2025.44015

**Published:** 2025-09-10

**Authors:** Johan M. Søbstad, Turid H. Sulen, Helge E. S. Pettersen, Grete May Engeseth, Lukas A. Hirschi, Camilla H. Stokkevåg

**Affiliations:** a Cancer Clinic, Haukeland University Hospital, Bergen, Norway; b Department of Life Sciences and Health, Oslo Metropolitan University, Olso, Norway; c Department of Physics and Technology, University of Bergen, Bergen, Norway

**Keywords:** radiotherapy, artificial intelligence, contouring, auto-segmentation, organs at risk, head and neck cancer

## Abstract

**Background and purpose:**

Ensuring the reliability and accuracy of artificial intelligence (AI)-generated contours is paramount, as discrepancies could lead to inadequate protection of healthy tissues. With increasing clinical workload, the aim of this study was to assess the time-saving potential of AI-assisted organs at risk (OAR) contouring in head and neck cancer (HNC) treatment planning, while also evaluating geometric accuracy, variability, and dosimetric impact.

**Patient/material and methods:**

Twenty patients had 12 OAR contoured by 11 certified dosimetrists and ARTplan (Therapanacea), including the brainstem, cochleas, larynx, mandible, oral cavity, parotid glands, pharynx constrictor muscles, spinal cord, right submandibular gland and thyroid gland. Comparisons were made using geometrical metrics, including Mean Surface Distance, Dice Similarity Coefficient (DSC), Hausdorff Distance, Volume Difference, and Centre of Mass Difference, as well as relevant dose-volume metrics, and total contouring time.

**Results:**

Median manual contouring time of the OARs was 55 (range: 17–151) minutes per patient, while adjusted AI-based structures required 17 (7–42), resulting in 69% time saved. For manual, adjusted and AI-contours, the mean DSC were generally high, averaging 0.85, 0.86, and 0.81 respectively across the evaluated structures. Notably, variability was lowest for the AI and adjusted contours. Average mean and max dose differences were acceptably low (<3.2 Gy) for all OARs.

**Interpretation:**

The results support the integration of AI-based contouring in HNC treatment planning. With minor adjustments, the contours achieve very good clinical quality and demonstrate improved consistency compared to manual contours, while significantly reducing contouring time.

## Graphical abstract

**Figure:**
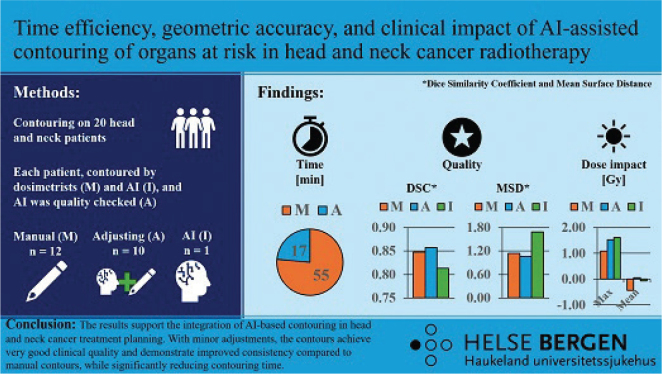

## Introduction

As cancer treatments become increasingly advanced, modern radiotherapy places increasing emphasis on achieving precise tumor targeting while minimizing radiation exposure to surrounding healthy tissue [1]. This shift has led to a growing need for accurate delineation of numerous organs at risk (OARs), a process that is essential for optimizing treatment efficacy and patient safety [2]. However, the expanding number of structures requiring contouring has significantly increased the workload for clinicians and highlighted the need for more efficient and standardized solutions [3]. In response, artificial intelligence (AI) has been progressively integrated into the clinical workflow in recent years [4].

AI-based segmentation leverages advanced machine learning algorithms, particularly convolutional neural networks, to identify and delineate anatomical structures from medical images with high accuracy [5]. These systems are trained on large datasets of annotated images, allowing them to learn and generalize the complex patterns associated with different organs [5–7]. The rising demand on contouring not only leads to increased workload for practitioners, but also heightens the risk of variability and potential errors in the contouring process [8]. Inconsistent delineation can result in suboptimal treatment plans, negatively impacting clinical outcomes and reducing the accuracy of research studies and follow-up assessments [2]. AI-based tools have been shown to not only reduce time associated with manual delineation but also enhancing consistency and reproducibility of contouring across patients and practitioners [9–11].

Several studies have investigated the potential consequences of AI-based contouring. As summarized by Mackay et al. [12] existing literature is often limited to assessing one or a few isolated evaluation aspects. These may include geometrical analysis, time saving, dosimetric impact, qualitative assessment, or variability [8, 12]. For head and neck (HN), studies such as those by Doolan et al. and Guo et al. [13, 14] assessed several HN OARs, but limited their scope to geometrical analysis and either dosimetrical or time saving analysis. Similar studies include that by Smine et al. [15] which investigated automatic segmentation for breast cancer. Additionally, our study includes an adjusted AI control group, representing the clinically relevant scenario in which AI-generated contours are routinely reviewed and modified. Other studies, such as those by Sarria et al. [16], conducted a study assessing AI-assisted contouring using one experienced clinician for both delineation and quality control, offering valuable data on time savings and dosimetric consistency, albeit without including variation. Given the substantial variability in delineation [2, 3], it remains a key factor to consider when evaluating the clinical applicability of AI-assisted contouring tools. The primary aim of this study was to assess the time-saving potential of AI-assisted OAR contouring in head and neck cancer (HNC) treatment planning. Secondary objectives included evaluating the geometric accuracy, variability, and dosimetric impact of AI-generated contours before and after manual adjustment.

## Material and methods

Twenty previously treated HNC patients were included in this study. In the cohort, four patients had cancer in the oral cavity, 13 in the pharynx (oropharynx, nasopharynx, hypopharynx), and three at other sites (larynx and lymph nodes). Five of the patients had surgery prior to treatment, removing their left submandibular gland. In terms of elective neck irradiation, 13 patients had bilateral target volumes, six patients had unilateral target volumes, and one patient had none. Each patient had 12 relevant OAR selected, including the brainstem, both cochlea, larynx, mandible, oral cavity, both parotid glands, pharynx constrictor muscles (PCMs), spinal cord (SC), right submandibular gland, and the thyroid gland. All OARs were present in at least 16 patients. Although 12 OARs were evaluated, cochleas were excluded from the geometrical evaluations due to substantial relative size discrepancies between AI and manual contours, which distorted figure scaling. They are, however, included in the time and dose analyses, where these discrepancies did not affect the presentation of results.

Each of the 20 patients were furthermore delineated independently by 11 certified dosimetrists. In total, 2,629 contours were segmented manually (20 patients × 12 OARs × 11 delineators, minus 11 exceptions). Details regarding exceptions are found in Supplementary material D. All had completed internal training and certification in manual OAR contouring, according to department-specific quality-assured guidelines [2, 17, 18]. All 12 OARs were delineated for all 20 patients, onwards termed the ‘manual group’ of contours. The ground truth (GT) contours were established by one experienced dosimetrist (not included in the manual contouring group). These initial contours were then sequentially peer reviewed by two dosimetrists, each correcting the preceding version according to clinical practice, without averaging or consensus voting. Both peer reviewers were previously included in the manual contouring group before reviewing the GT independently. Finally, a medical physicist reviewed the contours with a specific focus on correcting artefacts in the mandible. All contouring was performed in our treatment management system Aria^TM^ (Varian medical systems, Palo Alto) using our standard contouring tools, including brush/eraser, slice interpolation, and imaging threshold. A chart of the contouring workflow is found in Supplementary material D.

The patient set was furthermore sent to the AI-contouring software ARTplan™ v2.1 (Therapanacea, Paris) for segmentation and imported to Aria. All AI-generated contours are reviewed prior to clinical use; accordingly, a quality-assured set, ‘Adjusted group’, was created with manual adjustments made in select cases. In total, 1,980 contours were adjusted (20 patients × 10 OARs × 10 delineators, minus 20 exceptions). Adjustments were performed by 10 individuals, including those involved in the GT consensus and most members of the manual group.

A volume modulated arc therapy (VMAT) photon treatment plan, with prescription typically 2 Gy × 33–34 fractions was generated for each patient based on the OARs from the GT and the original clinical target volumes. The plans were made in the treatment planning system (TPS) Eclipse (Varian Medical Systems) and the dose distribution was used for the dosimetrical evaluation. During planning, OAR objectives were based on the recommendations from the DAHANCA trial [19] and our clinical procedures for the target volumes.

To assess the quality and efficiency of the contouring, geometrical, dosimetrical, and time metrics were considered. For the geometrical evaluation, we calculated the Mean Surface Distance (MSD), Dice Similarity Coefficient (DSC), Hausdorff Distance (HD), Volume Difference (VD), and Centre of Mass Difference (CMD) for each structure against the GT [12]. The dosimetrical evaluation assessed differences in dose across the various structure sets, focusing on clinically relevant dose metrics. Variability in structures was quantified as the standard deviation (SD) across all observers and all patients for each OAR.

Time used per OAR was measured during manual contouring. This was done by timing each delineation separately, rounded off to the closest half minute to simplify the registration process. As for timing the quality assurance and potential adjustment of the structure set from ART, the time for the complete set was recorded in one sitting. Timing the processing time for ART, as well as the import time (to the TPS) was done for the first nine patients only.

An in-house Python script was created to calculate the geometrical metrics. Each structure was converted to a binary volumetric mask using skimage.draw.polygon and numpy, and subject to geometrical calculations using the medpy functions for DSC (dc), HD (hd) and MSD (assd). For the calculation of CMD, scipy.ndimage.centre_of_mass was employed on the 3D binary mask. Similarly, numpy.sum was used to calculate the volume and thus the VD between the two binary objects. In medpy, the DSC is calculated as the weighted intersect between the two binary objects, DSC = 2|𝐴 ∩ 𝐵| ⁄ (|𝐴| + |𝐵|); the HD is calculated as the symmetric maximum surface distance between the binary objects, found using the Eucledian distance transform (EDT) of scipy.ndimage on the eroded surfaces; and the MSD is calculated similarly as the HD, but using the average distance from the EDT instead of the maximum. Regarding the SC, a caudal cut-off was applied at the slice where the first structure in the comparison ends, such that all SCs for the respective patients end at that slice, including the GT, AI-originals, the manual structures, and the adjusted structures. The SC was manually contoured on CT using a vertebral window level of -20 to 100 Hounsfield units (HU), segmenting visible cord at regular intervals, followed by interpolation and manual correction as needed.

To compare the relevant dose metrics, we collected all the structures per patient into one structure set. Another python script was employed to extract the Eclipse-calculated Dmean, Dmin, Dmedian, and Dmax parameters from the exported DVH files, finally transforming them into a tabular format using pandas in Python. Maximum dose values were reported as the absolute maximum dose within the structure, as provided by the TPS. With the default dose grid resolution of 0.25 cm, this corresponds to a voxel volume of 0.0156 cc.

This report is written using the SQUIRE 2.0 guidelines as base framework [20].

## Results

The median processing and import time for AI contouring was 10 minutes (range: 7–21), with average time at 11 minutes and 27 seconds. For the further estimates of time saved, 10 minutes were used as baseline expected processing and import time.

**Figure 1:**
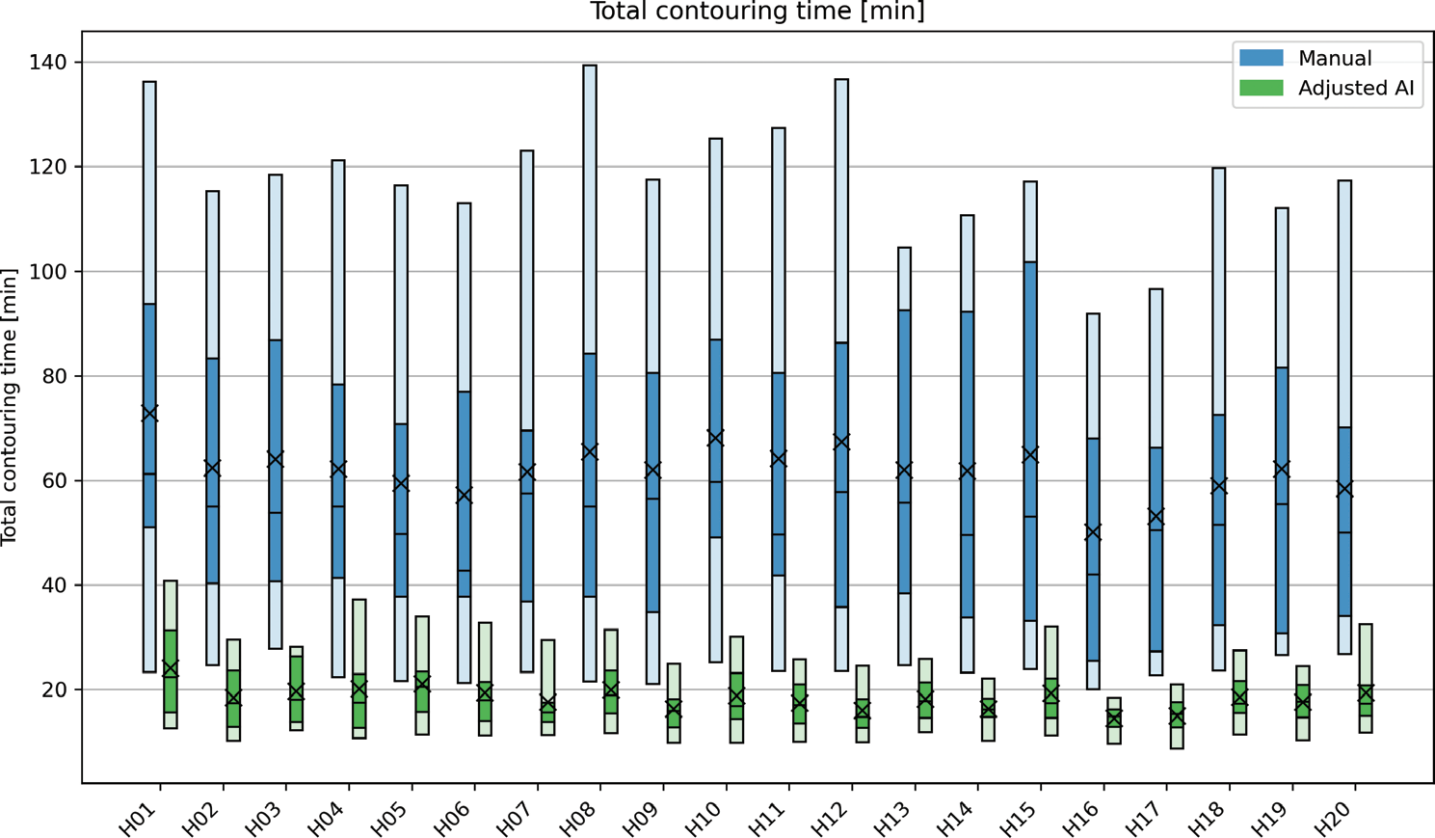
Time [minutes] used during manual and adjusted AI-based* contouring of the selected OARs per patient (H1-20). The blue columns represent the time used for manual contouring, while the green columns show the time used for controlling and adjusting of the AI-based contours, AI processing time excluded. The plot shows the 5-25-50-75-95 percentiles and the mean (X) value of each group. *Cochlea’s were manually contoured only and are included in both series. AI: artificial intelligence; OAR: organs at risk.

The time needed for manual contouring of each patient is shown in Figure 1. Overall, the median manual contouring time was 55 minutes (range: 17–151) and average time was 61.90 ± 33.75 minutes. The time needed for reviewing and adjusting the AI-contouring required a median time of 17 minutes (range: 7–42) and average at 18.38 ± 6.89 minutes. The median time saved was thereby approximately 38 minutes (–69%) or an average of 43.52 ± 34.45 minutes. For all cases, the worst 25th percentile ‘adjusted’ times were lower than the best 25th percentile of ‘manual contouring’ times. When accounting for processing and imports, the median time saved was approximately 28 minutes (–51%). Time measurement values are detailed in Supplementary material A.

**Figure 2:**
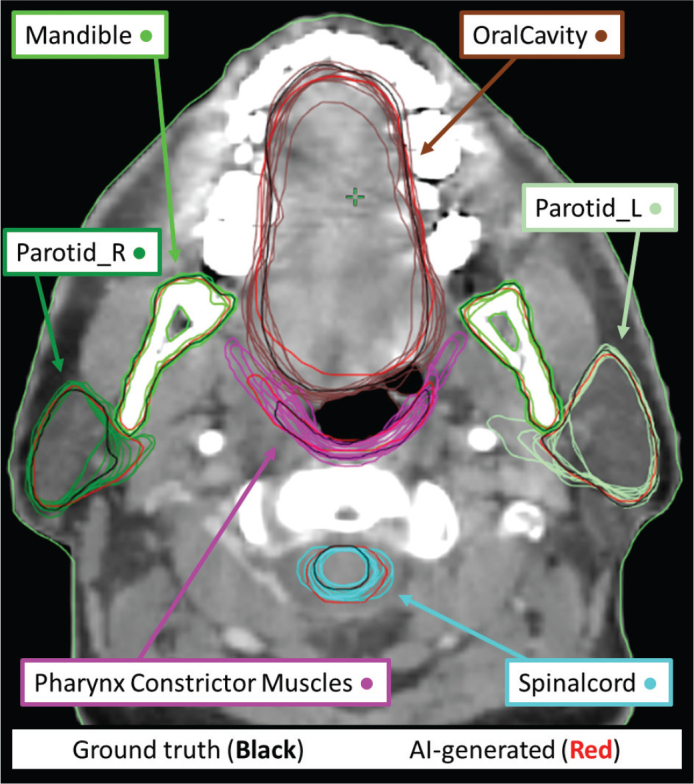
Example slice of one head and neck patient. The figure shows the ground truth in black, the AI-based structures in red, and the various manual contours of the relevant organs and their inter-observer variations. AI: artificial intelligence.

**Figure 3:**
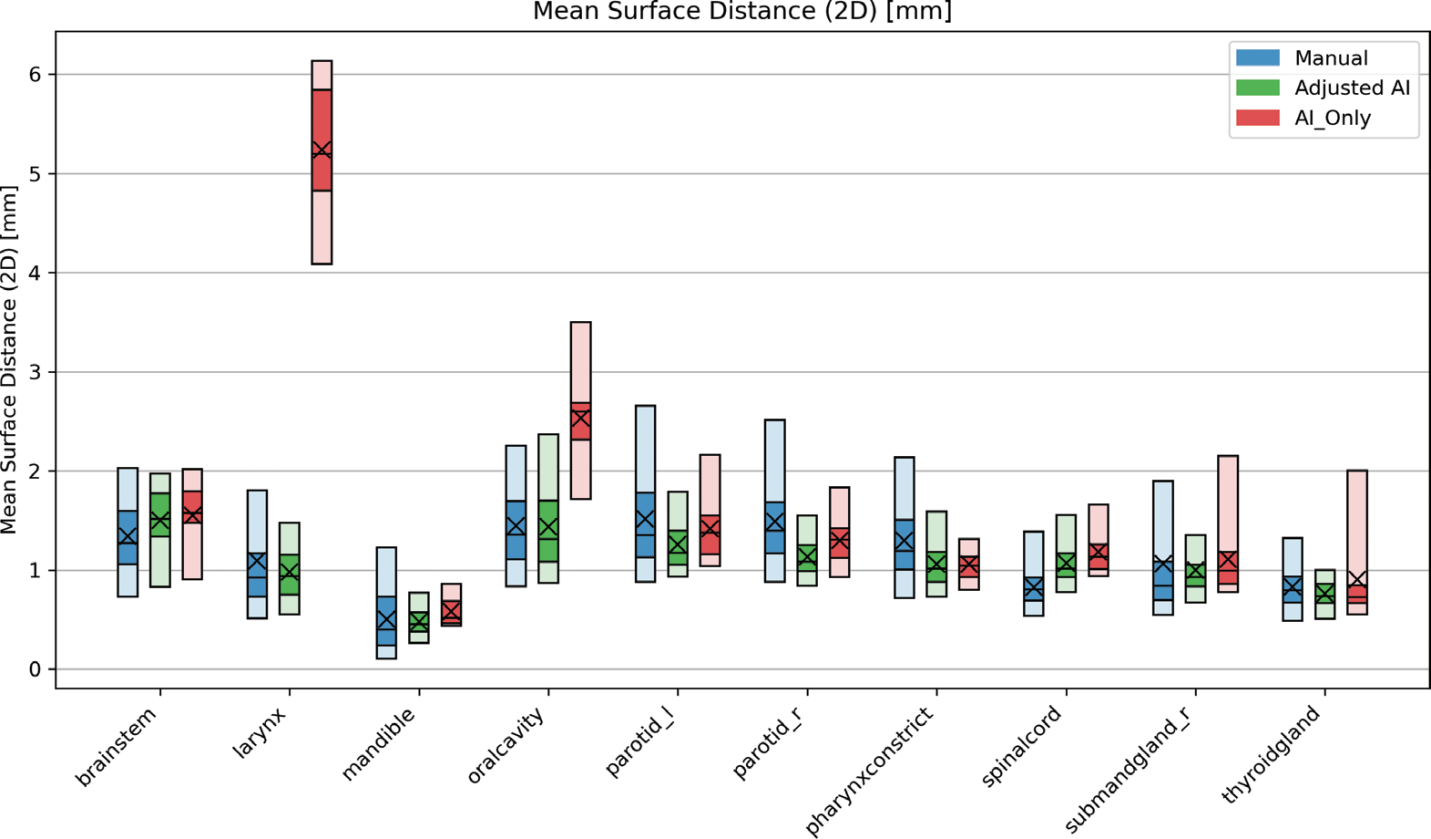
Results [mm] when calculating the Mean Surface Distance between the contour groups and the ground truth for all 20 patients. Zero indicates no difference between the surface of the GT and the structure. The blue bars contain the results of the manual contours (total n = 2,629), while the AI contours that were adjusted (total n = 1,980) and AI-originals (total n = 238) are green and red, respectively. The plot shows the 5-25-50-75-95 percentiles and the mean (X) value of each group. AI: artificial intelligence; GT: ground truth.

**Figure 4:**
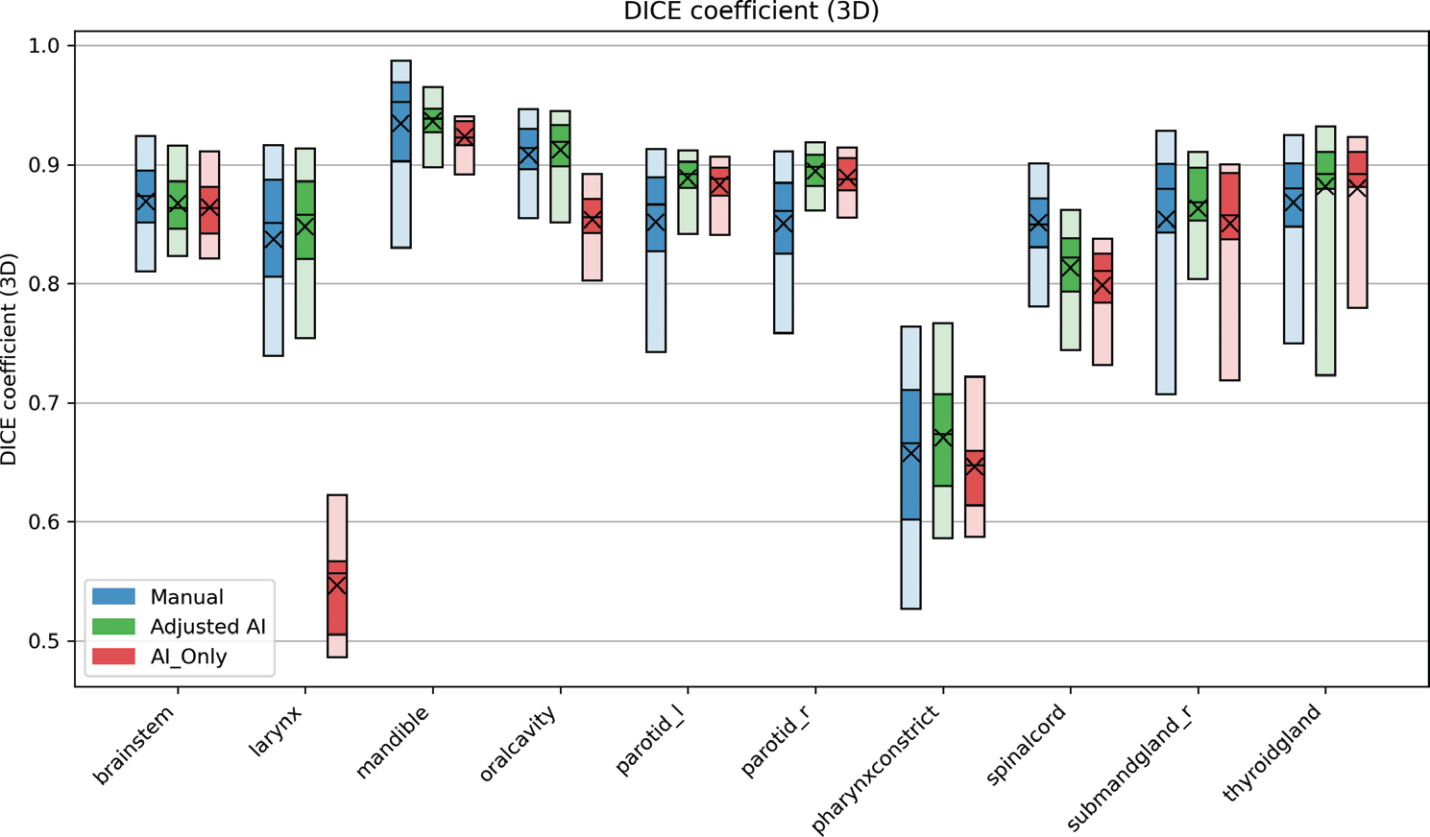
Results when calculating the Dice similarify coefficient between the contour groups and the ground truth for all 20 patients. A result of 1 indicates no difference between the surface of the GT and the structure. The blue bars contain the results of the manual contours (total n = 2,629), while the AI contours that were adjusted (total n = 1,980) and AI-originals (total n = 238) are green and red, respectively. The plot shows the 5-25-50-75-95 percentiles and the mean (X) value of each group. AI: artificial intelligence; GT: ground truth.

**Figure 5:**
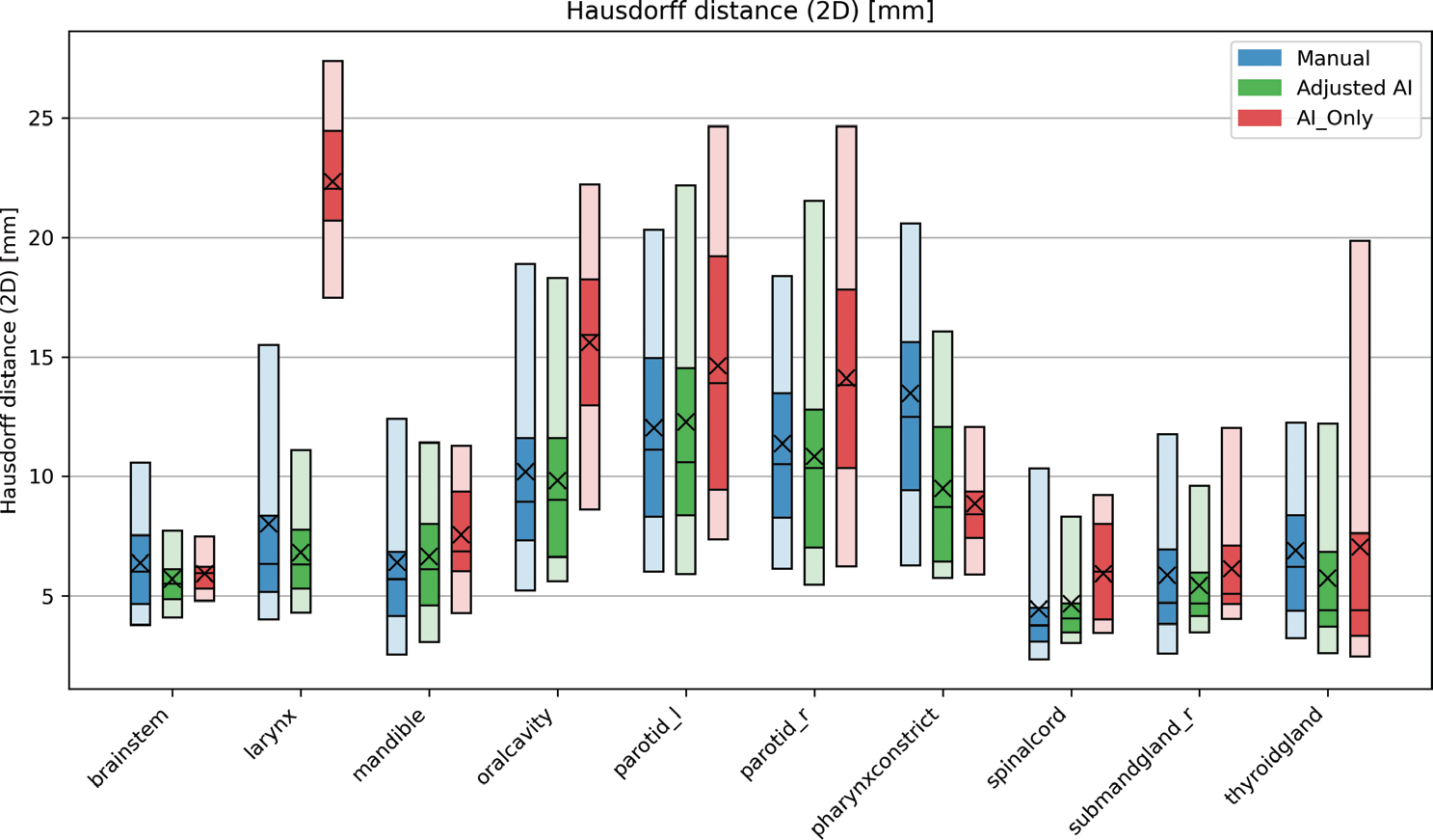
Results [mm] when calculating the Hausdorff distance between the contour groups and the ground truth for all 20 patients. Zero indicates no difference between the surface of the GT and the structure. The blue bars contain the results of the manual contours (total n = 2,629), while the AI contours that were adjusted (total n = 1,980) and AI-originals (total n = 238) are green and red, respectively. The plot shows the 5-25-50-75-95 percentiles and the mean (X) value of each group. AI: artificial intelligence; GT: ground truth.

The results from the geometric evaluation can be found in Figures 2–5, with values detailed in Supplementary material B. The variability is exemplified in Figure 2. The overall variations across contours in the geometric results for the OARs in terms of SD were reduced by 36–50% for the adjusted group and 27–47% for the AI only group, depending on the geometric evaluation. For MSD the mean SD values were 0.587 mm, 0.293 mm (–50%), and 0.380 mm (–35%) for the manual, adjusted, and AI_Only groups, respectively. DSC mean SD values were reduced from 0.064 to 0.036 (–44%) and 0.034 (–47%), HD from 5.317 to 3.069 mm (–42%) and 3.632 mm (–32%), VD from 4.846cc to 3.026cc (–38%) and 3.193cc (–34%), and CMD from 1.742 to 1.112 mm (–36%) and 1.275 mm (–27%).

For the DSC, results were mostly above 0.8 as seen in Figure 4. We also observed that there was a large discrepancy in contouring the PCM, with results mostly in the 0.6–0.7 region. The mandible had the best mean DSC results of 0.93 ± 0.05, 0.94 ± 0.02, and 0.92 ± 0.02, for the manual, adjusted AI, and AI_Only groups, respectively.

For the MSD, the majority of results were <2 mm (Figure 3). The mandible had the best mean MSD results of 0.50 ± 0.34 mm, 0.48 ± 0.15 mm, and 0.58 ± 0.15 mm for the manual, adjusted AI, and AI_Only groups, respectively.

For the HD (Figure 5), we observed that most structure groups had results over 10 mm. The left parotid had a mean HD of 12.03 ± 4.83 mm, 12.28 ± 5.31 mm, and 14.62 ± 6.05 mm, for the manual, adjusted AI, and AI_Only groups, respectively, while the right parotid gland had a mean HD of 11.37 ± 4.37 mm, 10.84 ± 4.86 mm, and 14.11 ± 5.76 mm.

The absolute VD is shown in Supplementary Figure 1 and relative VD in Supplementary Figure 2. Most results were within 10 cubic centimeters (cc) of the GT. The right submandibular gland had the best overall VD results, with a mean VD of –0.33 ± 1.66cc, 0.11 ± 1.51cc, and 0.03 ± 1.69cc for the manual, adjusted AI, and AI_Only groups, respectively.

The CMD were within 2–4 mm (Supplementary Figure 3), with the exceptions of the AI’s larynx, oral cavity, and the PCM from all groups. The thyroid had the overall best results with a mean CMD of 1.12 ± 0.92 mm, 0.93 ± 0.69 mm, and 1.10 ± 0.93 mm for the manual, adjusted AI, and AI_Only groups, respectively.

**Figure 6:**
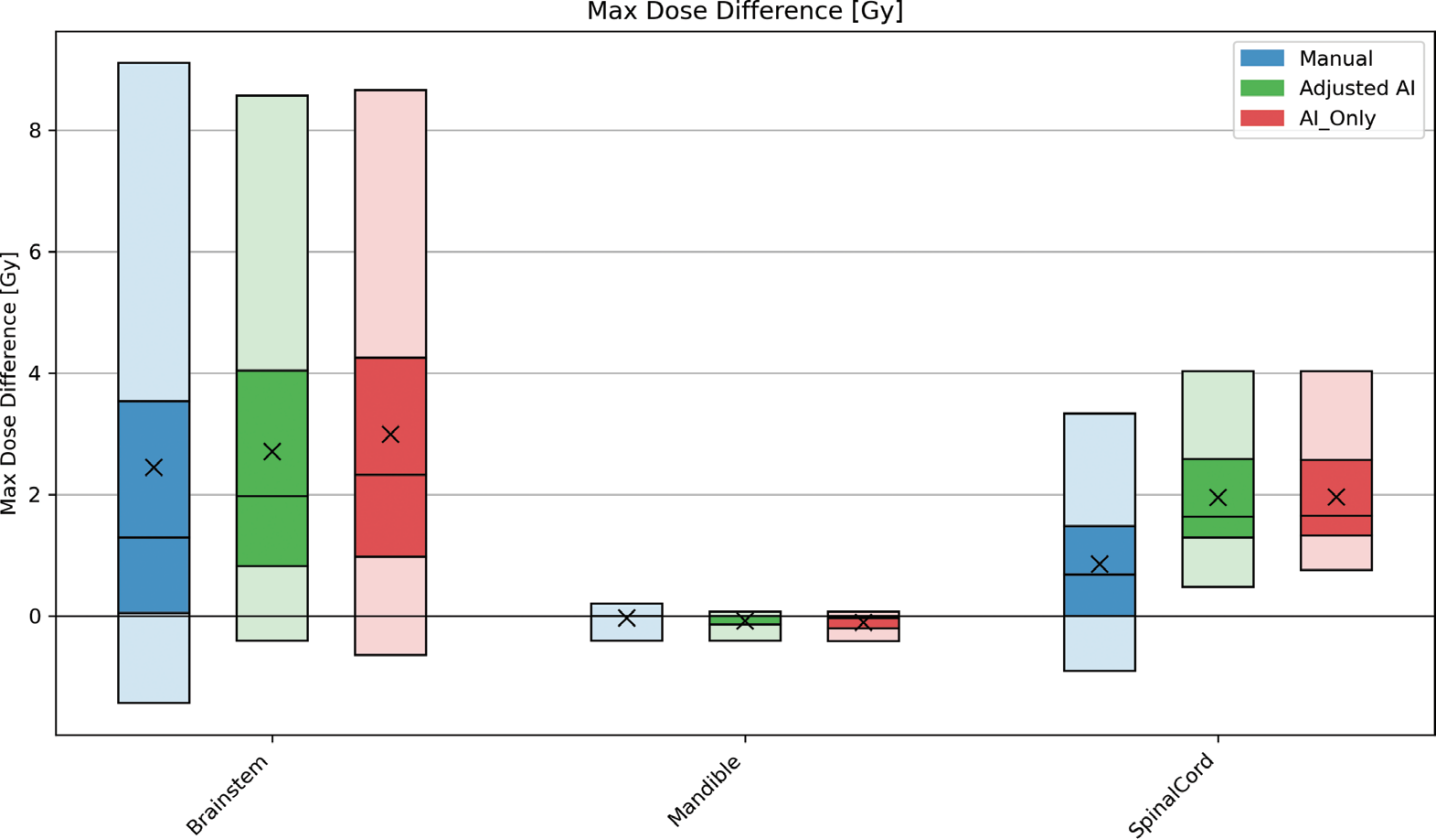
Results when calculating the difference in maximum dose [Gy] between the contour groups and the ground truth for all 20 patients. The blue bars contain the results of the manual contours, while the AI contours that were adjusted and AI-originals are green and red, respectively. The plot shows the 5-25-50-75-95 percentiles and the mean (X) value of each group. AI: artificial intelligence.

**Figure 7:**
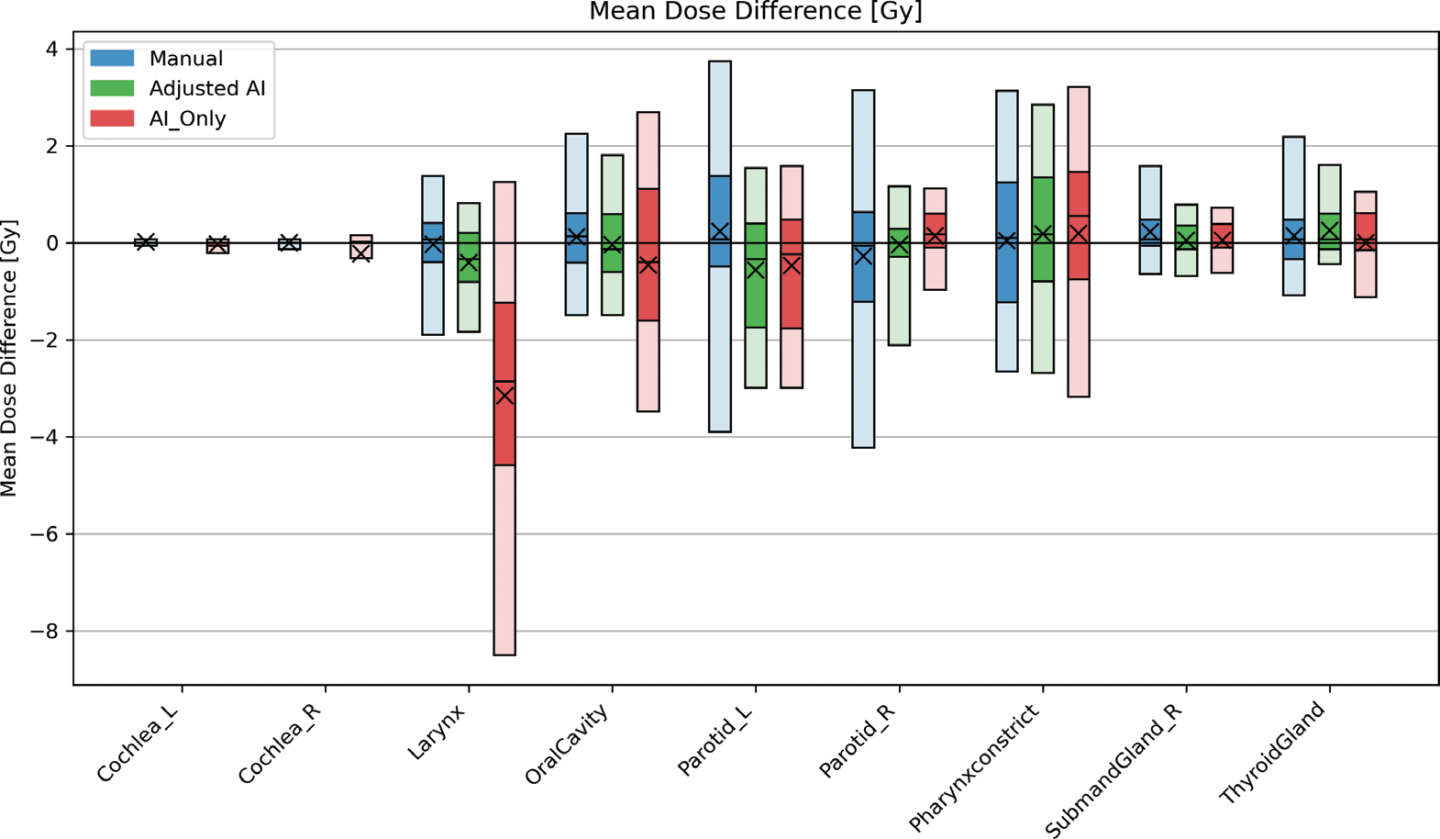
Results when calculating the difference in mean dose [Gy] between the contour groups and the ground truth for all 20 patients. The blue bars contain the results of the manual contours, while the AI contours that were adjusted and AI-originals are green and red, respectively. The plot shows the 5-25-50-75-95 percentiles and the mean (X) value of each group.

The results from the dosimetric evaluation can be found in Figures 6 and 7, with values detailed in Supplementary material C. Max dose difference between the groups and GT was evaluated for the serial organs, as well as the mandible, per our standard clinical goals. The brainstem had an average max dose difference of 2.45 ± 3.89 Gy, 2.71 ± 2.76 Gy, and 2.99 ± 3.12 Gy for the manual, adjusted AI, and AI_Only groups, respectively.

Mean dose difference between the groups and GT was evaluated for the remaining, parallel organs, per our standard clinical goals. The left parotid had an average mean dose difference of 0.23 ± 2.10 Gy, –0.56 ± 1.47 Gy, and –0.48 ± 1.66 Gy for the manual, adjusted AI, and AI_Only groups, respectively.

## Discussion and conclusion

Implementation of AI-assisted contouring in HN radiotherapy yielded substantial workflow benefits. Manual contouring required a median of 55 minutes per case, whereas review and adjustment of AI-generated contours took just 17 minutes. Even when including AI processing and imports, the net reduction was 28 minutes (–51%). Compared to the GT, most OARs demonstrated good agreement, with strong overlap and minimal surface discrepancies. Variability decreased, and dose-VDs compared to manual contours remained within clinically acceptable thresholds.

While we obtained a net time reduction of 51%, earlier evaluations of a previous software release of the same vendor reported time savings up to 95%, likely reflecting differences in OAR selection, and notably their exclusion of the larynx [13]. On the contrary, a multi-institutional study across seven global sites, involving 11 lymph node levels and seven OARs, reported time savings of up to 49% [21]. A study involving breast cancer patients reported time savings of at least 50% [15].

Our results showed that variability was notably reduced in the AI-assisted group compared to manual delineations. This improvement likely reflects the stabilizing effect of having a consistent baseline contour provided by the AI, which can help guide user corrections and reduce subjective variation. Similar improvements were reported by Pang et al. in their multicenter evaluation, where AI-generated contours led to significantly lower variability across institutions and users [21].

The geometric analyses confirmed robust contour accuracy. DSCs exceeded 0.8 for most OARs, with MSDs typically under 2 mm, which is comparable to prior studies [8, 9, 22–24]. Exceptions included the larynx (AI-only series) and pharyngeal constrictor muscles (PCM) in all arms, where some cases fell into the intermediate accuracy range. This is consistent with prior findings showing MSDs up to 2 mm for these complex muscles [8, 24], which likely amplifies small contouring discrepancies into larger relative errors. HDs were below 15 mm for most structures, consistent with previous deep learning studies in HN radiotherapy [8, 21, 25]. The SC’s elevated HD values likely reflect a guideline discrepancy in defining the brainstem–cord junction. Parotid glands exhibited large HD deviations (>15–20 mm) across all arms, similar to some prior findings [22, 26]. This was probably due to inconsistent delineation of their small anterior lobes by both AI and clinicians; these lobes minimally affect volume-based metrics but significantly influence surface–distance measures. Interestingly, the AI often yielded lower HD for the pharyngeal constrictors compared to manual and adjusted contours, suggesting superior AI delineation of their anterior extensions. We note that alternative distance metrics such as the 95th percentile HD are commonly used and may provide complementary information, particularly in mitigating sensitivity to outlier voxels and thin extensions.

Volume analysis revealed that for some AI-generated contours, such as the brainstem and SC, the defined diameters were larger than in manual contours; however, these increases were largely retained after clinician correction. Structures such as the brainstem and SC required only minor adjustments, reinforcing the AI’s baseline reliability. Most CMD deviations across OARs were limited to 2–3 mm, indicating strong spatial agreement. Unexpectedly, spinal-cord CMD shifts reached 3–4 mm in some instances, a potentially significant displacement when targets lie close to this critical structure. In clinical practice, MRI confirmation in borderline cases would likely reduce localization errors and variability. A longitudinal-axis CMD analysis at the brainstem–cord junction could further elucidate systematic trends but was beyond the current study’s scope.

Mean dose differences across all OARs (except the larynx) were typically less than 1–2 Gy, with max dose differences slightly higher for the brainstem and SC due to AI’s marginally expanded contours. These findings align with those of a study in 10 nasopharyngeal carcinoma patients, where deep-learning auto-segmentation showed no significant impact on most OAR doses [14]. Furthermore, large geometric discrepancies in the cochleas had negligible effect on mean dose when targets lay sufficiently distant and may justify omission of an adjusted AI arm for this structure.

Despite the improvements seen in the adjusted group compared to the AI-only contours, the magnitude of change in geometric metrics such as the median DSC was modest for several OARs. These observations combined with a mostly negligible difference in dose, suggest the potential for a ‘quick review’ or omission of selected structures to further streamline the contouring process. Notably, many of the corrections involved minor boundary refinements rather than substantial structural changes, which may have limited impact on overlap-based metrics like DSC. In anatomically complex or ambiguous regions, however, manual refinement may still provide critical improvements in local accuracy not fully captured by these metrics, which could be especially important for high-risk or target-adjacent OARs. These findings highlight both the robustness of the AI model and the case-dependent value of clinician oversight, reinforcing the importance of maintaining a thoughtful and clinically guided review process.

Of our 2,629 manually delineated OARs, two were entirely misplaced, but remained in the analysis. This rare (<0.1%) human error underscores the value of automated quality checks. Occasional substantial deviations in expert manual contours have been documented, reinforcing the case for integrated AI-based workflows [1]. No unexpected gross errors were observed in the AI outputs beyond known guideline differences.

A limitation of this study is that repeated delineations were performed on the same set of patients, introducing dependency across observations. This study did not include certain routinely contoured structures (e.g. left submandibular gland, lips, esophagus), but their inclusion in future work could broaden applicability. A detailed axial center of mass analysis and AI correction for the cochleas, despite minimal dosimetric impact, also warrant further investigation. The GT was reviewed by dosimetrists who also contributed to the manual and adjusted-AI groups, which could introduce reviewer bias. Furthermore, consensus was formed without external validation or averaging, and all reviewers were from the same institution. No treatment plan re-optimization was performed using the compared structure sets. The dosimetric differences therefore reflect plans originally optimized using GT OARs and may not fully represent the clinical impact that could result from re-optimizing with alternative OAR sets.

To conclude, this work is among the first to simultaneously assess time savings, geometric performance, dosimetric differences, and variability related to AI-assisted contouring in a cohort of HNC patients. AI-assisted contours achieved substantially reduced delineation time, while providing clinical-grade quality, enhanced consistency, with negligible dosimetric impact. These advantages support the integration of such AI-tools into routine radiotherapy workflows, offering a promising path toward improved efficiency and standardization.

## Supplementary Material









## Data Availability

Data available upon request.

## References

[1] Harari PM, Song S, Tomé WA. Emphasizing conformal avoidance versus target definition for IMRT planning in head-and-neck cancer. Int J Radiat Oncol Biol Phys. 2010;77(3):950–8. 10.1016/j.ijrobp.2009.09.062 20378266 PMC2905233

[2] Brouwer CL, Steenbakkers RJ, Bourhis J, Budach W, Grau C, Grégoire V, et al. CT-based delineation of organs at risk in the head and neck region: DAHANCA, EORTC, GORTEC, HKNPCSG, NCIC CTG, NCRI, NRG Oncology and TROG consensus guidelines. Radiother Oncol. 2015;117(1):83–90. 10.1016/j.radonc.2015.07.041 26277855

[3] Boero IJ, Paravati AJ, Xu B, Cohen EE, Mell LK, Le Q-T, et al. Importance of radiation oncologist experience among patients with head-and-neck cancer treated with intensity-modulated radiation therapy. J Clin Oncol. 2016;34(7):684–90 10.1200/JCO.2015.63.9898 26729432 PMC4872027

[4] Vrtovec T, Močnik D, Strojan P, Pernuš F, Ibragimov B. Auto‐segmentation of organs at risk for head and neck radiotherapy planning: from atlas‐based to deep learning methods. Med Phys. 2020;47(9):e929–50 10.1002/mp.14320 32510603

[5] Harrison K, Pullen H, Welsh C, Oktay O, Alvarez-Valle J, Jena R. Machine learning for auto-segmentation in radiotherapy planning. Clin Oncol. 2022;34(2):74–88. 10.1016/j.clon.2021.12.003 34996682

[6] Cardenas CE, Yang J, Anderson BM, Court LE, Brock KB. Advances in auto-segmentation. Semin Radiat Oncol. 2019;29(3):185–97. 10.1016/j.semradonc.2019.02.001 31027636

[7] Claessens M, Oria CS, Brouwer CL, Ziemer BP, Scholey JE, Lin H, et al. Quality assurance for AI-based applications in radiation therapy. Semin Radiat Oncol. 2022;32(4):421–31. 10.1016/j.semradonc.2022.06.011 36202444

[8] Kosmin M, Ledsam J, Romera-Paredes B, Mendes R, Moinuddin S, De Souza D, et al. Rapid advances in auto-segmentation of organs at risk and target volumes in head and neck cancer. Radiother Oncol. 2019;135:130–40. 10.1016/j.radonc.2019.03.004 31015159

[9] Walker GV, Awan M, Tao R, Koay EJ, Boehling NS, Grant JD, et al. Prospective randomized double-blind study of atlas-based organ-at-risk autosegmentation-assisted radiation planning in head and neck cancer. Radiother Oncol. 2014;112(3):321–5. 10.1016/j.radonc.2014.08.028 25216572 PMC4252740

[10] Tao C-J, Yi J-L, Chen N-Y, Ren W, Cheng J, Tung S, et al. Multi-subject atlas-based auto-segmentation reduces interobserver variation and improves dosimetric parameter consistency for organs at risk in nasopharyngeal carcinoma: a multi-institution clinical study. Radiother Oncol. 2015;115(3):407–11. 10.1016/j.radonc.2015.05.012 26025546

[11] Peters LJ, O’Sullivan B, Giralt J, Fitzgerald TJ, Trotti A, Bernier J, et al. Critical impact of radiotherapy protocol compliance and quality in the treatment of advanced head and neck cancer: results from TROG 02.02. J Clin Oncol. 2010;28(18):2996–3001. 10.1200/JCO.2009.27.4498 20479390

[12] Mackay K, Bernstein D, Glocker B, Kamnitsas K, Taylor A. A review of the metrics used to assess auto-contouring systems in radiotherapy. Clin Oncol. 2023;35(6):354–69. 10.1016/j.clon.2023.01.016 36803407

[13] Doolan PJ, Charalambous S, Roussakis Y, Leczynski A, Peratikou M, Benjamin M, et al. A clinical evaluation of the performance of five commercial artificial intelligence contouring systems for radiotherapy. Front Oncol. 2023;13:1213068 10.3389/fonc.2023.1213068 37601695 PMC10436522

[14] Guo H, Wang J, Xia X, Zhong Y, Peng J, Zhang Z, et al. The dosimetric impact of deep learning-based auto-segmentation of organs at risk on nasopharyngeal and rectal cancer. Radiat Oncol. 2021;16(1):113. 10.1186/s13014-021-01837-y 34162410 PMC8220801

[15] Smine Z, Poeta S, De Caluwé A, Desmet A, Garibaldi C, Boni KB, et al. Automated segmentation in planning-CT for breast cancer radiotherapy: a review of recent advances. J Radiother Oncol. 2025;202:110615. 10.1016/j.radonc.2024.110615 39489430

[16] Sarria G, Kugel F, Layer J, Dejonckheere C, Scafa D, Roehner F, et al. AI-based auto-segmentation: advantages in delineation, absorbed dose-distribution and logistics. Adv Radiat Oncol. 2023;9(3):101394. 10.1016/j.adro.2023.101394 38292888 PMC10823084

[17] Mir R, Kelly SM, Xiao Y, Moore A, Clark CH, Clementel E, et al. Organ at risk delineation for radiation therapy clinical trials: Global Harmonization Group consensus guidelines. Radiother Oncol. 2020;150:30–9. 10.1016/j.radonc.2020.05.038 32504762

[18] Bisello S, Cilla S, Benini A, Cardano R, Nguyen NP, Deodato F, et al. Dose–volume constraints fOr oRganS At risk In Radiotherapy (CORSAIR): an ‘all-in-one’ multicenter–multidisciplinary practical summary. Curr Oncol. 2022;29(10):7021–50. 10.3390/curroncol29100552 36290829 PMC9600677

[19] Jensen K, Friborg J, Hansen CR, Samsøe E, Johansen J, Andersen M, et al. The Danish head and neck cancer group (DAHANCA) 2020 radiotherapy guidelines. Radiother Oncol. 2020;151:149–51. 10.1016/j.radonc.2020.07.037 32781011

[20] Ogrinc G, Davies L, Goodman D, Batalden P, Davidoff F, Stevens D. SQUIRE 2.0 (S tandards for QU ality I mprovement R eporting E xcellence): revised publication guidelines from a detailed consensus process. J Contin Educ Nurs. 2015;46(11):501–7. 10.3928/00220124-20151020-02 26509402

[21] Pang EPP, Tan HQ, Wang F, Niemelä J, Bolard G, Ramadan S, et al. Multicentre evaluation of deep learning CT autosegmentation of the head and neck region for radiotherapy. NPJ Digit Med. 2025;8(1):312. 10.1038/s41746-025-01624-z 40419731 PMC12106707

[22] Fritscher KD, Peroni M, Zaffino P, Spadea MF, Schubert R, Sharp G. Automatic segmentation of head and neck CT images for radiotherapy treatment planning using multiple atlases, statistical appearance models, and geodesic active contours. Med Phys. 2014;41(5):051910. 10.1118/1.4871623 24784389 PMC4000401

[23] Ibragimov B, Xing L. Segmentation of organs‐at‐risks in head and neck CT images using convolutional neural networks. Med Phys. 2017;44(2):547–57. 10.1002/mp.12045 28205307 PMC5383420

[24] Teguh DN, Levendag PC, Voet PW, Al-Mamgani A, Han X, Wolf TK, et al. Clinical validation of atlas-based auto-segmentation of multiple target volumes and normal tissue (swallowing/mastication) structures in the head and neck. Int J Radiat Oncol Biol Phys. 2011;81(4):950–7. 10.1016/j.ijrobp.2010.07.009 20932664

[25] Van Dijk LV, Van den Bosch L, Aljabar P, Peressutti D, Both S, Steenbakkers RJ, et al. Improving automatic delineation for head and neck organs at risk by deep learning contouring. Radiother Oncol. 2020;142:115–23. 10.1016/j.radonc.2019.09.022 31653573

[26] Fritscher K, Raudaschl P, Zaffino P, Spadea MF, Sharp GC, Schubert R, editors. Deep neural networks for fast segmentation of 3D medical images. Medical Image Computing and Computer-Assisted Intervention–MICCAI 2016: 19th International Conference; 2016 October 17–21; Athens, Proceedings, Part II 19. Springer; 2016. 10.1007/978-3-319-46723-8_19

