# Supplementary material for: Time efficiency, geometric accuracy, and clinical impact of AI-assisted contouring of organs at risk in head and neck cancer radiotherapy
Source: Acta Oncol. 2025 Sep 10;64:44015. doi: 10.2340/1651-226X.2025.44015 (PMC12439213; doi:10.2340/1651-226X.2025.44015)
Supplement: Supplementary file 1 [file AO-64-44015-s1.pdf]

Supplementary material has been published as submitted. It has not been copyedited, or typeset by Acta Oncologica

Supplementary material A – Time measurement details

Supplementary Table 1: Mean and standard deviation values [min] of the manual and adjusted contour groups per patient. Includes percentiles that were shown in the results.

Metric	Patient	Series	Mean + SD	5 %	25 %	50 %	75 %	95 %
Total time [min]	Head01	Manual	72.82±40.08	23.38	51.00	61.20	93.75	136.25
Total time [min]	Head01	Adjusted	24.12±10.82	12.64	15.63	22.38	31.38	40.73
Total time [min]	Head02	Manual	62.32±32.54	24.65	40.30	55.00	83.25	115.35
Total time [min]	Head02	Adjusted	18.47±7.35	10.22	12.90	17.40	23.65	29.60
Total time [min]	Head03	Manual	64.02±33.68	27.78	40.65	53.75	86.75	118.45
Total time [min]	Head03	Adjusted	19.69±6.70	12.19	13.81	18.06	26.31	28.11
Total time [min]	Head04	Manual	62.18±34.27	22.42	41.30	55.00	78.25	121.15
Total time [min]	Head04	Adjusted	20.13±9.93	10.70	12.73	17.48	22.98	37.23
Total time [min]	Head05	Manual	59.48±34.16	21.65	37.80	49.76	70.75	116.35
Total time [min]	Head05	Adjusted	21.07±8.09	11.33	15.73	20.48	23.48	34.03
Total time [min]	Head06	Manual	57.15±32.70	21.30	37.80	42.78	77.00	112.95
Total time [min]	Head06	Adjusted	19.34±7.88	11.19	13.98	17.98	21.48	32.78
Total time [min]	Head07	Manual	61.64±34.34	23.30	36.80	57.50	69.50	123.00
Total time [min]	Head07	Adjusted	17.61±6.67	11.27	13.80	15.62	17.47	29.47
Total time [min]	Head08	Manual	65.50±40.82	21.52	37.80	55.00	84.25	139.35
Total time [min]	Head08	Adjusted	19.94±7.48	11.61	15.39	18.89	23.64	31.44
Total time [min]	Head09	Manual	62.01±35.92	21.04	34.80	56.50	80.50	117.50
Total time [min]	Head09	Adjusted	16.27±5.70	9.88	12.76	15.90	18.15	24.90
Total time [min]	Head10	Manual	68.08±36.69	25.26	49.06	59.70	86.88	125.30
Total time [min]	Head10	Adjusted	18.79±7.53	9.85	14.36	16.86	23.11	30.11
Total time [min]	Head11	Manual	64.17±38.37	23.55	41.80	49.68	80.50	127.35
Total time [min]	Head11	Adjusted	17.36±5.90	10.09	13.50	17.00	21.00	25.80
Total time [min]	Head12	Manual	67.33±40.71	23.54	35.80	57.74	86.25	136.70
Total time [min]	Head12	Adjusted	16.03±5.43	9.93	12.73	14.81	18.18	24.61
Total time [min]	Head13	Manual	62.01±30.91	24.62	38.40	55.78	92.50	104.50
Total time [min]	Head13	Adjusted	18.18±5.19	11.84	14.59	17.81	21.31	25.86
Total time [min]	Head14	Manual	61.76±33.96	23.21	33.80	49.50	92.25	110.70
Total time [min]	Head14	Adjusted	16.32±4.15	10.29	14.74	16.14	18.24	22.14
Total time [min]	Head15	Manual	64.90±37.12	23.92	33.15	53.00	101.75	117.12
Total time [min]	Head15	Adjusted	19.27±7.58	11.22	14.61	17.36	22.11	32.01
Total time [min]	Head16	Manual	50.10±27.38	20.00	25.55	42.00	68.00	91.85
Total time [min]	Head16	Adjusted	14.47±3.15	9.70	12.85	14.85	16.15	18.45
Total time [min]	Head17	Manual	53.07±27.42	22.81	27.30	50.47	66.25	96.57
Total time [min]	Head17	Adjusted	14.97±4.39	8.70	12.77	15.34	17.60	20.99
Total time [min]	Head18	Manual	58.93±35.18	23.64	32.25	51.50	72.50	119.72
Total time [min]	Head18	Adjusted	18.55±5.83	11.33	15.48	17.35	21.60	27.45
Total time [min]	Head19	Manual	62.18±33.23	26.62	30.80	55.50	81.62	112.02
Total time [min]	Head19	Adjusted	17.59±5.22	10.36	14.64	17.64	20.89	24.49

Total time [min]	Head20	Manual	58.36±32.73	26.80	34.05	50.00	70.12	117.28
Total time [min]	Head20	Adjusted	19.36±7.51	11.77	14.92	17.32	20.82	32.52
