# Supplementary material for: Time efficiency, geometric accuracy, and clinical impact of AI-assisted contouring of organs at risk in head and neck cancer radiotherapy
Source: Acta Oncol. 2025 Sep 10;64:44015. doi: 10.2340/1651-226X.2025.44015 (PMC12439213; doi:10.2340/1651-226X.2025.44015)
Supplement: Supplementary file 2 [file AO-64-44015-s2.pdf]

Supplementary material has been published as submitted. It has not been copyedited, or typeset by Acta Oncologica

Supplementary material B – Geometric details

Supplementary Table 2: Table with all geometric metrics (Volume Difference (VD), Centre of Mass Difference (CMD), Dice Similarity Coefficient (DSC), Hausdorff Distance (HD) and Mean Surface Distance (MSD)), including the mean with standard deviation and various percentiles that were shown in the results. AI: artificial intelligence.

Metric	OAR	Series	Mean + SD	5 %	25 %	50 %	75 %	95 %
VD [cc]	brainstem	Manual	0.28±3.92	-7.12	-2.26	0.64	2.96	6.03
VD [cc]	brainstem	Adjusted	4.86±2.40	1.20	3.23	4.90	6.59	8.67
VD [cc]	brainstem	AI_Only	5.03±2.42	0.95	3.57	5.42	6.56	8.39
VD [cc]	larynx	Manual	-1.09±4.97	-6.97	-3.24	-1.12	0.50	4.53
VD [cc]	larynx	Adjusted	-0.04±3.73	-5.88	-2.44	0.25	1.75	5.22
VD [cc]	larynx	AI_Only	20.28±4.82	12.47	17.37	19.91	23.23	27.71
VD [cc]	mandible	Manual	-3.03±7.82	-19.5	-6.28	-0.98	1.96	5.98
VD [cc]	mandible	Adjusted	-3.35±3.25	-8.20	-5.29	-3.58	-1.77	2.74
VD [cc]	mandible	AI_Only	-3.86±2.62	-7.59	-5.08	-4.03	-2.57	0.36
VD [cc]	oralcavity	Manual	1.56±10.90	-13.16	-4.97	0.76	8.13	19.4
VD [cc]	oralcavity	Adjusted	0.28±8.72	-14.42	-4.28	0.19	5.39	12.9
VD [cc]	oralcavity	AI_Only	-12.55±10.17	-28.67	-19.09	-8.83	-6.47	0.03
VD [cc]	parotid_l	Manual	-3.12±4.84	-12.20	-6.09	-2.83	0.20	4.15
VD [cc]	parotid_l	Adjusted	-3.25±2.58	-7.39	-5.19	-3.37	-1.42	0.77
VD [cc]	parotid_l	AI_Only	-4.05±2.56	-8.18	-5.78	-3.85	-2.42	0.10
VD [cc]	parotid_r	Manual	-3.06±4.62	-11.20	-6.46	-2.62	0.22	4.68
VD [cc]	parotid_r	Adjusted	-1.75±1.77	-4.17	-3.04	-2.06	-0.73	1.74
VD [cc]	parotid_r	AI_Only	-2.48±1.87	-5.69	-3.64	-2.23	-1.51	0.38
VD [cc]	pharynxconstrict	Manual	-0.11±4.34	-7.25	-2.78	0.51	2.55	6.33
VD [cc]	pharynxconstrict	Adjusted	-0.29±2.22	-5.11	-1.04	0.07	1.25	2.69
VD [cc]	pharynxconstrict	AI_Only	-1.05±2.09	-5.25	-1.82	-0.46	0.51	1.06
VD [cc]	spinalcord	Manual	0.33±2.31	-3.46	-1.26	0.23	1.83	4.34
VD [cc]	spinalcord	Adjusted	3.65±1.62	0.93	2.7	3.47	4.63	6.54
VD [cc]	spinalcord	AI_Only	3.70±1.60	1.38	2.53	3.47	4.63	6.33
VD [cc]	submandgland_r	Manual	-0.33±1.66	-2.95	-1.19	-0.29	0.53	1.76
VD [cc]	submandgland_r	Adjusted	0.11±1.51	-2.12	-0.59	0.43	1.10	1.91
VD [cc]	submandgland_r	AI_Only	0.03±1.69	-3.61	-0.35	0.52	0.96	1.74
VD [cc]	thyroidgland	Manual	0.24±3.08	-5.42	-1.27	0.35	2.11	4.66
VD [cc]	thyroidgland	Adjusted	0.65±2.46	-4.85	-0.65	0.97	1.70	5.48
VD [cc]	thyroidgland	AI_Only	0.91±2.09	-2.82	0.03	0.68	1.31	4.98
CMD [mm]	brainstem	Manual	1.80±1.35	0.47	0.90	1.48	2.29	4.08
CMD [mm]	brainstem	Adjusted	1.44±0.72	0.52	0.88	1.36	1.85	3.05
CMD [mm]	brainstem	AI_Only	1.27±0.55	0.54	0.79	1.21	1.61	2.26
CMD [mm]	larynx	Manual	1.72±2.08	0.37	0.76	1.18	1.89	4.16
CMD [mm]	larynx	Adjusted	1.57±1.21	0.51	0.92	1.34	2.01	2.86
CMD [mm]	larynx	AI_Only	12.67±2.10	9.05	11.01	12.64	13.55	15.41

CMD [mm]	mandible	Manual	1.16±0.97	0.15	0.49	0.89	1.46	2.85
CMD [mm]	mandible	Adjusted	1.44±0.85	0.29	0.87	1.26	1.96	3.15
CMD [mm]	mandible	AI_Only	2.41±0.93	1.02	1.87	2.40	3.04	3.8
CMD [mm]	oralcavity	Manual	1.79±1.54	0.43	0.97	1.40	2.15	4.21
CMD [mm]	oralcavity	Adjusted	1.89±1.16	0.51	1.05	1.62	2.38	4.29
CMD [mm]	oralcavity	AI_Only	4.37±1.45	2.28	3.42	4.42	5.03	6.99
CMD [mm]	parotid_l	Manual	2.15±1.32	0.61	1.11	1.75	3.00	4.81
CMD [mm]	parotid_l	Adjusted	1.61±0.72	0.43	1.11	1.59	2.06	2.88
CMD [mm]	parotid_l	AI_Only	1.84±0.76	0.77	1.34	1.74	2.36	2.99
CMD [mm]	parotid_r	Manual	2.10±1.29	0.55	1.15	1.79	2.71	5.04
CMD [mm]	parotid_r	Adjusted	1.51±0.69	0.59	1.00	1.40	1.95	2.94
CMD [mm]	parotid_r	AI_Only	1.90±0.76	0.81	1.35	1.95	2.41	3.06
CMD [mm]	pharynxconstrict	Manual	4.85±3.59	1.04	2.34	3.96	6.33	12.28
CMD [mm]	pharynxconstrict	Adjusted	4.37±2.66	0.84	2.13	4.11	5.95	8.81
CMD [mm]	pharynxconstrict	AI_Only	6.53±2.37	3.40	5.32	5.67	8.53	10.05
CMD [mm]	spinalcord	Manual	2.79±2.05	0.47	1.09	2.28	4.12	6.78
CMD [mm]	spinalcord	Adjusted	2.12±1.51	0.57	1.03	1.63	2.94	4.93
CMD [mm]	spinalcord	AI_Only	2.66±1.81	0.79	1.40	1.89	3.38	5.88
CMD [mm]	submandgland_r	Manual	1.51±2.31	0.22	0.61	0.96	1.67	3.74
CMD [mm]	submandgland_r	Adjusted	1.46±0.91	0.44	0.87	1.35	1.69	2.69
CMD [mm]	submandgland_r	AI_Only	1.87±1.09	0.72	1.34	1.50	2.01	4.77
CMD [mm]	thyroidgland	Manual	1.12±0.92	0.28	0.53	0.86	1.43	3.07
CMD [mm]	thyroidgland	Adjusted	0.93±0.69	0.28	0.52	0.68	1.06	2.93
CMD [mm]	thyroidgland	AI_Only	1.10±0.93	0.29	0.59	0.80	1.26	3.21
DSC	brainstem	Manual	0.87±0.04	0.81	0.85	0.87	0.89	0.92
DSC	brainstem	Adjusted	0.87±0.03	0.82	0.85	0.86	0.89	0.92
DSC	brainstem	AI_Only	0.86±0.03	0.82	0.84	0.86	0.88	0.91
DSC	larynx	Manual	0.84±0.09	0.74	0.81	0.85	0.89	0.92
DSC	larynx	Adjusted	0.85±0.05	0.75	0.82	0.86	0.89	0.91
DSC	larynx	AI_Only	0.55±0.04	0.49	0.51	0.56	0.57	0.62
DSC	mandible	Manual	0.93±0.05	0.83	0.90	0.95	0.97	0.99
DSC	mandible	Adjusted	0.94±0.02	0.90	0.93	0.94	0.95	0.97
DSC	mandible	AI_Only	0.92±0.02	0.89	0.92	0.92	0.94	0.94
DSC	oralcavity	Manual	0.91±0.05	0.86	0.90	0.91	0.93	0.95
DSC	oralcavity	Adjusted	0.91±0.03	0.85	0.90	0.92	0.93	0.94
DSC	oralcavity	AI_Only	0.85±0.03	0.80	0.84	0.86	0.87	0.89
DSC	parotid_l	Manual	0.85±0.06	0.74	0.83	0.87	0.89	0.91
DSC	parotid_l	Adjusted	0.89±0.02	0.84	0.88	0.89	0.90	0.91
DSC	parotid_l	AI_Only	0.88±0.02	0.84	0.87	0.89	0.90	0.91
DSC	parotid_r	Manual	0.85±0.05	0.76	0.83	0.86	0.88	0.91
DSC	parotid_r	Adjusted	0.89±0.02	0.86	0.88	0.90	0.91	0.92
DSC	parotid_r	AI_Only	0.89±0.02	0.86	0.88	0.89	0.91	0.91
DSC	pharynxconstrict	Manual	0.66±0.09	0.53	0.6	0.67	0.71	0.76
DSC	pharynxconstrict	Adjusted	0.67±0.05	0.59	0.63	0.67	0.71	0.77
DSC	pharynxconstrict	AI_Only	0.65±0.04	0.59	0.61	0.65	0.66	0.72

DSC	spinalcord	Manual	0.85±0.04	0.78	0.83	0.85	0.87	0.90
DSC	spinalcord	Adjusted	0.81±0.04	0.74	0.79	0.82	0.84	0.86
DSC	spinalcord	AI_Only	0.80±0.04	0.73	0.78	0.81	0.83	0.84
DSC	submandgland_r	Manual	0.85±0.11	0.71	0.84	0.88	0.90	0.93
DSC	submandgland_r	Adjusted	0.86±0.05	0.80	0.85	0.87	0.90	0.91
DSC	submandgland_r	AI_Only	0.85±0.05	0.72	0.84	0.86	0.89	0.90
DSC	thyroidgland	Manual	0.87±0.06	0.75	0.85	0.88	0.90	0.92
DSC	thyroidgland	Adjusted	0.88±0.05	0.72	0.88	0.89	0.91	0.93
DSC	thyroidgland	AI_Only	0.88±0.05	0.78	0.88	0.89	0.91	0.92
HD [mm]	brainstem	Manual	6.38±2.33	3.78	4.67	6.00	7.53	10.56
HD [mm]	brainstem	Adjusted	6.43±10.06	4.09	4.85	5.51	6.12	7.77
HD [mm]	brainstem	AI_Only	5.93±0.90	4.79	5.30	5.95	6.21	7.49
HD [mm]	larynx	Manual	8.01±9.85	4.00	5.17	6.33	8.35	15.49
HD [mm]	larynx	Adjusted	6.82±2.40	4.29	5.32	6.31	7.76	11.11
HD [mm]	larynx	AI_Only	22.33±3.10	17.47	20.71	22.04	24.46	27.39
HD [mm]	mandible	Manual	6.41±5.43	2.54	4.15	5.69	6.83	12.41
HD [mm]	mandible	Adjusted	6.64±2.86	3.06	4.61	6.11	8.00	11.42
HD [mm]	mandible	AI_Only	7.55±2.41	4.27	6.03	6.85	9.37	11.29
HD [mm]	oralcavity	Manual	10.19±4.63	5.23	7.33	8.93	11.6	18.9
HD [mm]	oralcavity	Adjusted	9.82±3.96	5.62	6.62	9.02	11.59	18.3
HD [mm]	oralcavity	AI_Only	15.60±4.26	8.61	12.99	15.93	18.25	22.22
HD [mm]	parotid_l	Manual	12.03±4.83	6.00	8.33	11.13	14.95	20.33
HD [mm]	parotid_l	Adjusted	12.28±5.31	5.91	8.38	10.58	14.53	22.18
HD [mm]	parotid_l	AI_Only	14.62±6.05	7.38	9.46	13.91	19.21	24.65
HD [mm]	parotid_r	Manual	11.37±4.37	6.13	8.29	10.51	13.48	18.39
HD [mm]	parotid_r	Adjusted	10.84±4.86	5.47	7.02	10.36	12.8	21.55
HD [mm]	parotid_r	AI_Only	14.11±5.76	6.24	10.36	13.82	17.82	24.66
HD [mm]	pharynxconstrict	Manual	13.48±11.62	6.29	9.44	12.48	15.61	20.58
HD [mm]	pharynxconstrict	Adjusted	9.49±3.41	5.74	6.45	8.71	12.08	16.08
HD [mm]	pharynxconstrict	AI_Only	8.86±2.05	5.88	7.44	8.42	9.38	12.08
HD [mm]	spinalcord	Manual	4.45±2.67	2.35	3.09	3.76	4.50	10.35
HD [mm]	spinalcord	Adjusted	4.67±1.84	3.02	3.47	4.05	4.69	8.32
HD [mm]	spinalcord	AI_Only	5.93±2.14	3.44	4.00	6.00	8.00	9.22
HD [mm]	submandgland_r	Manual	5.87±3.81	2.59	3.81	4.70	6.94	11.76
HD [mm]	submandgland_r	Adjusted	5.44±2.07	3.47	4.15	4.69	5.96	9.61
HD [mm]	submandgland_r	AI_Only	6.14±2.38	4.03	4.66	5.08	7.11	12.03
HD [mm]	thyroidgland	Manual	6.91±3.63	3.22	4.38	6.22	8.39	12.26
HD [mm]	thyroidgland	Adjusted	5.74±2.79	2.61	3.72	4.41	6.83	12.21
HD [mm]	thyroidgland	AI_Only	7.04±7.27	2.46	3.32	4.41	7.62	19.86
MSD [mm]	brainstem	Manual	1.34±0.49	0.73	1.06	1.27	1.59	2.02
MSD [mm]	brainstem	Adjusted	1.50±0.36	0.83	1.33	1.51	1.77	1.97
MSD [mm]	brainstem	AI_Only	1.55±0.35	0.91	1.47	1.57	1.79	2.02
MSD [mm]	larynx	Manual	1.09±0.99	0.51	0.73	0.93	1.17	1.80
MSD [mm]	larynx	Adjusted	0.98±0.42	0.55	0.75	0.94	1.15	1.48
MSD [mm]	larynx	AI_Only	5.24±0.66	4.09	4.82	5.20	5.84	6.13

MSD [mm]	mandible	Manual	0.50±0.34	0.11	0.24	0.40	0.73	1.23
MSD [mm]	mandible	Adjusted	0.48±0.15	0.26	0.38	0.45	0.57	0.77
MSD [mm]	mandible	AI_Only	0.58±0.15	0.44	0.46	0.52	0.69	0.86
MSD [mm]	oralcavity	Manual	1.44±0.61	0.84	1.11	1.36	1.69	2.26
MSD [mm]	oralcavity	Adjusted	1.43±0.49	0.87	1.08	1.31	1.70	2.37
MSD [mm]	oralcavity	AI_Only	2.53±0.51	1.71	2.31	2.59	2.68	3.50
MSD [mm]	parotid_l	Manual	1.52±0.60	0.88	1.13	1.35	1.78	2.65
MSD [mm]	parotid_l	Adjusted	1.25±0.30	0.93	1.05	1.17	1.39	1.79
MSD [mm]	parotid_l	AI_Only	1.42±0.39	1.04	1.15	1.38	1.55	2.16
MSD [mm]	parotid_r	Manual	1.49±0.53	0.88	1.16	1.39	1.68	2.51
MSD [mm]	parotid_r	Adjusted	1.13±0.23	0.84	0.99	1.08	1.25	1.55
MSD [mm]	parotid_r	AI_Only	1.29±0.25	0.93	1.12	1.31	1.42	1.83
MSD [mm]	pharynxconstrict	Manual	1.29±0.52	0.71	1.00	1.19	1.50	2.14
MSD [mm]	pharynxconstrict	Adjusted	1.06±0.26	0.73	0.88	1.01	1.18	1.59
MSD [mm]	pharynxconstrict	AI_Only	1.06±0.16	0.80	0.93	1.04	1.13	1.31
MSD [mm]	spinalcord	Manual	0.82±0.26	0.53	0.69	0.81	0.93	1.39
MSD [mm]	spinalcord	Adjusted	1.07±0.23	0.77	0.93	1.01	1.17	1.55
MSD [mm]	spinalcord	AI_Only	1.18±0.24	0.94	1.01	1.13	1.26	1.66
MSD [mm]	submandgland_r	Manual	1.06±1.22	0.55	0.70	0.84	1.08	1.90
MSD [mm]	submandgland_r	Adjusted	1.00±0.34	0.67	0.84	0.93	1.05	1.35
MSD [mm]	submandgland_r	AI_Only	1.11±0.41	0.77	0.86	0.99	1.18	2.15
MSD [mm]	thyroidgland	Manual	0.83±0.31	0.49	0.67	0.79	0.93	1.32
MSD [mm]	thyroidgland	Adjusted	0.76±0.15	0.51	0.67	0.74	0.86	1.00
MSD [mm]	thyroidgland	AI_Only	0.90±0.68	0.55	0.67	0.73	0.85	2.00

Absolute Volume Difference

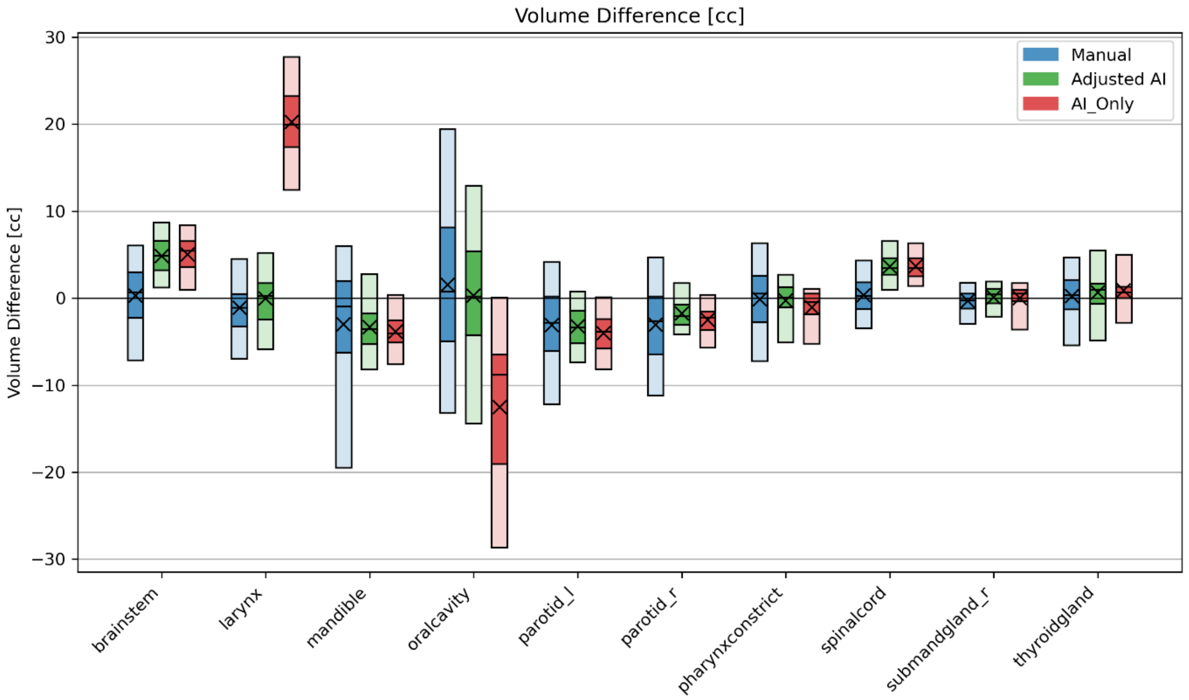

Supplementary Figure 1: Results when calculating the absolute volume difference between the contour groups and the ground truth for all twenty patients. The blue bars contain the results of the manual contours (total n = 2629), while the AI contours that were adjusted (total n = 1980) and AI-originals (total n = 238) are green and red, respectively. The plot shows the 5-25-50-75-95 percentiles and the mean (X) value of each group. AI: artificial intelligence.

Relative Volume Difference

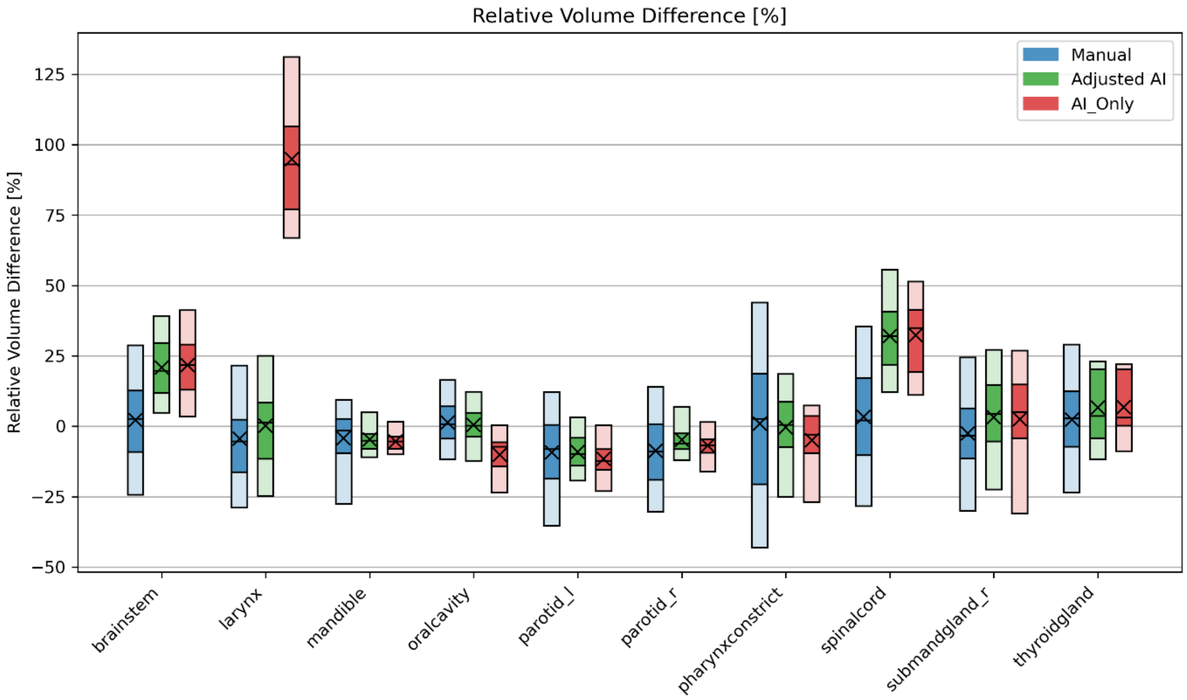

Supplementary Figure 2: Results when calculating the relative volume difference between the contour groups and the ground truth for all twenty patients. The blue bars contain the results of the manual contours (total n = 2629), while the AI contours that were adjusted (total n = 1980) and AI-originals (total n = 238) are green and red, respectively. The plot shows the 5-25-50-75-95 percentiles and the mean (X) value of each group. AI: artificial intelligence.

Supplementary Table 3: Table with Relative Volume Difference, including the mean [%] with standard deviation [%points] and various percentiles that were shown in Supplementary Figure 1. AI: artificial intelligence.

Metric	OAR	Series	Mean + SD	5 %	25 %	50 %	75 %	95 %
VD [%]	brainstem	Manual	2.20±16.09	16.09	-24.28	-9.10	2.55	12.64
VD [%]	brainstem	Adjusted	20.85±11.57	11.57	4.71	11.75	19.73	29.56
VD [%]	brainstem	AI_Only	21.72±12.13	12.13	3.51	12.98	21.63	28.97
VD [%]	larynx	Manual	-4.36±24.80	24.80	-28.89	-16.26	-5.46	2.39
VD [%]	larynx	Adjusted	0.22±16.80	16.80	-24.77	-11.61	1.30	8.34
VD [%]	larynx	AI_Only	94.89±22.17	22.17	66.81	77.01	92.94	106.51
VD [%]	mandible	Manual	-4.33±11.48	11.48	-27.62	-9.61	-1.48	2.67
VD [%]	mandible	Adjusted	-4.64±4.79	4.79	-10.94	-7.93	-5.18	-2.64
VD [%]	mandible	AI_Only	-5.42±3.87	3.87	-10.04	-8.00	-5.42	-3.65

VD [%]	oralcavity	Manual	1.32±9.86	9.86	-11.71	-4.40	0.70	7.00
VD [%]	oralcavity	Adjusted	0.43±7.40	7.40	-12.31	-3.75	0.18	4.76
VD [%]	oralcavity	AI_Only	-10.09±7.51	7.51	-23.42	-14.34	-7.30	-5.75
VD [%]	parotid_l	Manual	-9.25±14.96	14.96	-35.43	-18.57	-8.16	0.51
VD [%]	parotid_l	Adjusted	-9.18±7.08	7.08	-19.38	-14.07	-9.91	-4.13
VD [%]	parotid_l	AI_Only	-11.64±7.08	7.08	-22.99	-15.40	-12.26	-8.18
VD [%]	parotid_r	Manual	-8.75±13.59	13.59	-30.33	-19.05	-9.00	0.77
VD [%]	parotid_r	Adjusted	-4.84±5.57	5.57	-12.04	-8.19	-6.10	-2.61
VD [%]	parotid_r	AI_Only	-6.87±5.56	5.56	-16.14	-9.48	-6.84	-4.64
VD [%]	pharynxconstrict	Manual	0.96±27.68	27.68	-43.07	-20.66	2.69	18.72
VD [%]	pharynxconstrict	Adjusted	-0.37±12.97	12.97	-25.01	-7.35	0.43	8.68
VD [%]	pharynxconstrict	AI_Only	-4.96±11.22	11.22	-26.93	-9.57	-2.95	3.65
VD [%]	spinalcord	Manual	3.34±20.07	20.07	-28.29	-10.24	2.08	17.06
VD [%]	spinalcord	Adjusted	32.07±13.69	13.69	12.10	21.90	32.06	40.69
VD [%]	spinalcord	AI_Only	32.27±13.39	13.39	11.11	19.29	34.82	41.36
VD [%]	submandgland_r	Manual	-2.56±18.32	18.32	-30.05	-11.50	-3.36	6.40
VD [%]	submandgland_r	Adjusted	3.25±15.19	15.19	-22.42	-5.48	4.37	14.69
VD [%]	submandgland_r	AI_Only	2.44±16.63	16.63	-30.99	-4.28	5.08	14.81
VD [%]	thyroidgland	Manual	2.29±15.65	15.65	-23.49	-7.29	2.77	12.37
VD [%]	thyroidgland	Adjusted	6.57±12.31	12.31	-11.74	-4.26	3.57	20.26
VD [%]	thyroidgland	AI_Only	6.81±11.27	11.27	-8.92	0.11	3.10	20.26

Centre of Mass Difference

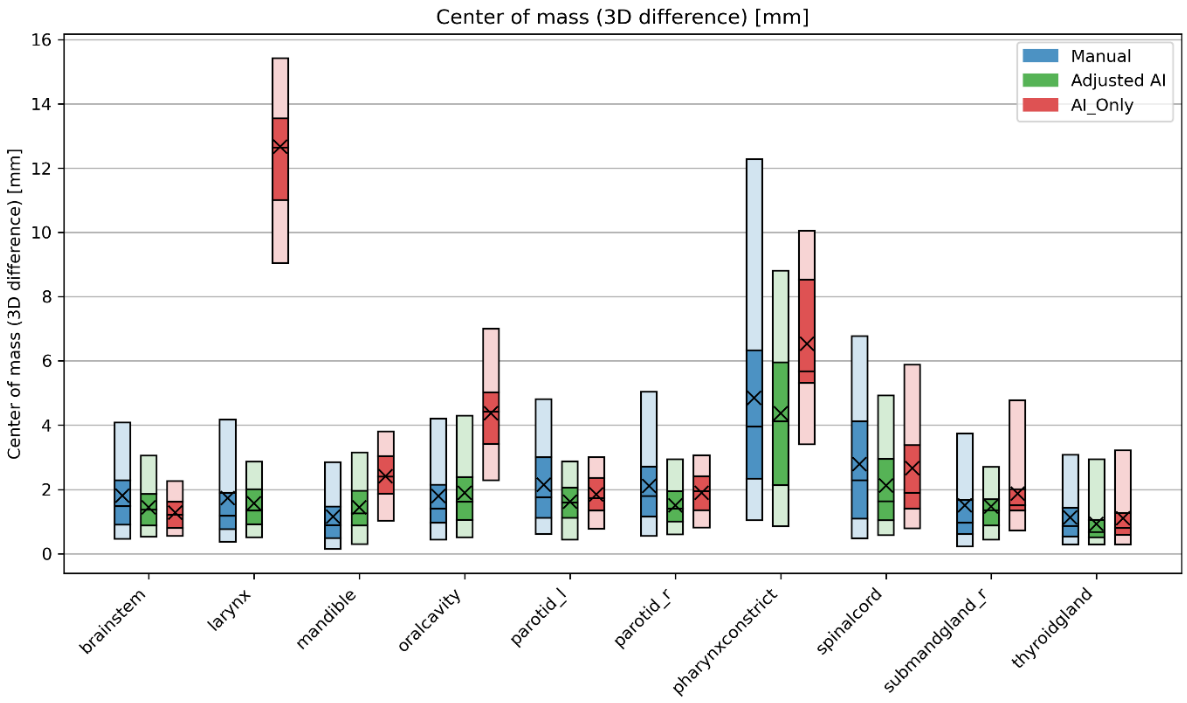

Supplementary Figure 3: Results when calculating the centre of mass difference between the contour groups and the ground truth for all twenty patients. The blue bars contain the results of the manual contours (total n = 2629), while the AI contours that were adjusted (total n =

1980) and AI-originals (total n = 238) are green and red, respectively. The plot shows the 5-25-50-75-95 percentiles and the mean (X) value of each group. AI: artificial intelligence.
