# Supplementary material for: Time efficiency, geometric accuracy, and clinical impact of AI-assisted contouring of organs at risk in head and neck cancer radiotherapy
Source: Acta Oncol. 2025 Sep 10;64:44015. doi: 10.2340/1651-226X.2025.44015 (PMC12439213; doi:10.2340/1651-226X.2025.44015)
Supplement: Supplementary file 3 [file AO-64-44015-s3.pdf]

Supplementary material has been published as submitted. It has not been copyedited, or typeset by Acta Oncologica

Supplementary material C – Dose metrics details

Supplementary Table 4: Table with max dose difference [Gy] details, including the mean with standard deviation and various percentiles that were shown in the results. AI: artificial intelligence. Dmax: D0.015625cc.

Metric	OAR	Series	Mean + SD	5 %	25 %	50 %	75 %	95 %
ΔDmax [Gy]	Brainstem	Manual	2.45±3.89	-1.44	0.05	1.29	3.54	9.10
ΔDmax [Gy]	Brainstem	Adjusted	2.71±2.76	-0.41	0.82	1.97	4.04	8.57
ΔDmax [Gy]	Brainstem	AI_Only	2.99±3.12	-0.64	0.97	2.33	4.25	8.66
ΔDmax [Gy]	Mandible	Manual	-0.03±0.24	-0.41	0.00	0.00	0.00	0.20
ΔDmax [Gy]	Mandible	Adjusted	-0.08±0.16	-0.41	-0.14	0.00	0.00	0.07
ΔDmax [Gy]	Mandible	AI_Only	-0.11±0.17	-0.41	-0.20	-0.03	0.00	0.07
ΔDmax [Gy]	SpinalCord	Manual	0.85±1.36	-0.90	0.00	0.68	1.48	3.33
ΔDmax [Gy]	SpinalCord	Adjusted	1.95±1.03	0.48	1.29	1.63	2.58	4.04
ΔDmax [Gy]	SpinalCord	AI_Only	1.96±1.02	0.75	1.33	1.65	2.57	4.04

Supplementary Table 5: Table with mean dose difference [Gy] details, including the mean with standard deviation and various percentiles that were shown in the results. The cochleas did not have an adjusted group in this study. AI: artificial intelligence.

Metric	OAR	Series	Mean + SD	5 %	25 %	50 %	75 %	95 %
ΔDmean [Gy]	Cochlea_L	Manual	0.02±0.16	-0.07	0.00	0.00	0.00	0.07
ΔDmean [Gy]	Cochlea_L	AI_Only	-0.04±0.11	-0.21	-0.06	0.00	0.00	0.06
ΔDmean [Gy]	Cochlea_R	Manual	0.00±0.23	-0.14	0.00	0.00	0.00	0.07
ΔDmean [Gy]	Cochlea_R	AI_Only	-0.23±1.17	-0.32	0.00	0.00	0.02	0.15
ΔDmean [Gy]	Larynx	Manual	-0.03±1.09	-1.91	-0.41	0.07	0.41	1.37
ΔDmean [Gy]	Larynx	Adjusted	-0.41±0.96	-1.84	-0.82	-0.34	0.20	0.82
ΔDmean [Gy]	Larynx	AI_Only	-3.16±3.18	-8.51	-4.58	-2.86	-1.24	1.25
ΔDmean [Gy]	OralCavity	Manual	0.12±1.79	-1.50	-0.42	0.13	0.61	2.24
ΔDmean [Gy]	OralCavity	Adjusted	-0.05±1.10	-1.50	-0.61	-0.14	0.58	1.80
ΔDmean [Gy]	OralCavity	AI_Only	-0.46±2.13	-3.49	-1.60	-0.41	1.10	2.69
ΔDmean [Gy]	Parotid_L	Manual	0.23±2.10	-3.90	-0.49	0.07	1.37	3.74
ΔDmean [Gy]	Parotid_L	Adjusted	-0.56±1.47	-2.99	-1.75	-0.34	0.39	1.54
ΔDmean [Gy]	Parotid_L	AI_Only	-0.48±1.66	-2.99	-1.77	-0.24	0.47	1.58
ΔDmean [Gy]	Parotid_R	Manual	-0.28±2.06	-4.23	-1.22	-0.06	0.63	3.13
ΔDmean [Gy]	Parotid_R	Adjusted	-0.05±0.88	-2.12	-0.29	0.00	0.28	1.16
ΔDmean [Gy]	Parotid_R	AI_Only	0.14±0.89	-0.97	-0.11	0.17	0.60	1.12
ΔDmean [Gy]	Pharynxconstrict	Manual	0.03±2.03	-2.66	-1.23	0.10	1.24	3.13
ΔDmean [Gy]	Pharynxconstrict	Adjusted	0.18±1.70	-2.69	-0.80	0.17	1.34	2.85
ΔDmean [Gy]	Pharynxconstrict	AI_Only	0.19±2.18	-3.18	-0.76	0.54	1.45	3.21
ΔDmean [Gy]	SubmandGland_R	Manual	0.22±0.71	-0.65	-0.07	0.07	0.48	1.58
ΔDmean [Gy]	SubmandGland_R	Adjusted	0.05±0.50	-0.69	-0.14	0.00	0.35	0.79
ΔDmean [Gy]	SubmandGland_R	AI_Only	0.05±0.54	-0.63	-0.10	0.00	0.38	0.72
ΔDmean [Gy]	ThyroidGland	Manual	0.14±1.17	-1.09	-0.34	0.07	0.48	2.18

ΔDmean [Gy]	ThyroidGland	Adjusted	0.25±0.61	-0.45	-0.14	0.07	0.60	1.60
ΔDmean [Gy]	ThyroidGland	AI_Only	-0.01±1.15	-1.13	-0.15	0.07	0.60	1.05
