# Supplementary material for: Time efficiency, geometric accuracy, and clinical impact of AI-assisted contouring of organs at risk in head and neck cancer radiotherapy
Source: Acta Oncol. 2025 Sep 10;64:44015. doi: 10.2340/1651-226X.2025.44015 (PMC12439213; doi:10.2340/1651-226X.2025.44015)
Supplement: Supplementary file 4 [file AO-64-44015-s4.pdf]

Supplementary material has been published as submitted. It has not been copyedited, or typeset by Acta Oncologica

Supplementary material D – Other information

Exceptions in the data

There were a few exceptions found in the data. For exceptions 1-4, time measurements were not corrected.

1. Patient H15 did not have a right submandibular gland at time of treatment. This structure is ignored for all structure sets of this patient.
2. ARTplan was not able to draw the pharynx constrictor muscles of patient H14. Therefore, this structure was not corrected by the dosimetrists and excluded from the data. It was, however, contoured manually and is included in the manual contour group.
3. During post-processing/data controlling some mistakes were found, such as missing interpolation (found=5) or structures not drawn (found=4). The missing interpolation were interpolated and corrected by our lead dosimetrist and assumed to be representative of what would have been drawn. The missing structures were contoured by the respective dosimetrist, but at a much later date than intended (several months). We assume that these are also representative of what would have been drawn during our initial round of contouring. However, it is entirely possible for the dosimetrist to have adapted slightly towards the artificial intelligence (AI) and thus the structures could be more similar. This is, however, a small sample of the total amount of structures.
4. During analysis, the adjusted brainstem of one dosimetrist on patient H17 was found to have a sub-voxel error that heavily skewed the Hausdorff Distance (HD) results for this patient. We chose to remove this HD result, while assuming this sub- voxel mistake has negligible consequences on the other geometric metrics.
5. Both cochleas, as well as the larynx are redrawn from scratch in the adjusted group. This is because our clinical experience indicates that such corrections would take longer than just redrawing. Hence, the cochleas were not drawn in the adjusted group. Instead, the time measured to draw the cochleas in the manual group were added to the adjusted group. The larynx was redrawn in the adjusted group and is therefore already included in the time measurements. Additionally, the cocheas were excluded from the geometric evaluation due to figure scaling distortion. This is because the cochleas are very small and will therefore amplify the relative measurements of the geometric evaluations.

Contouring workflow chart

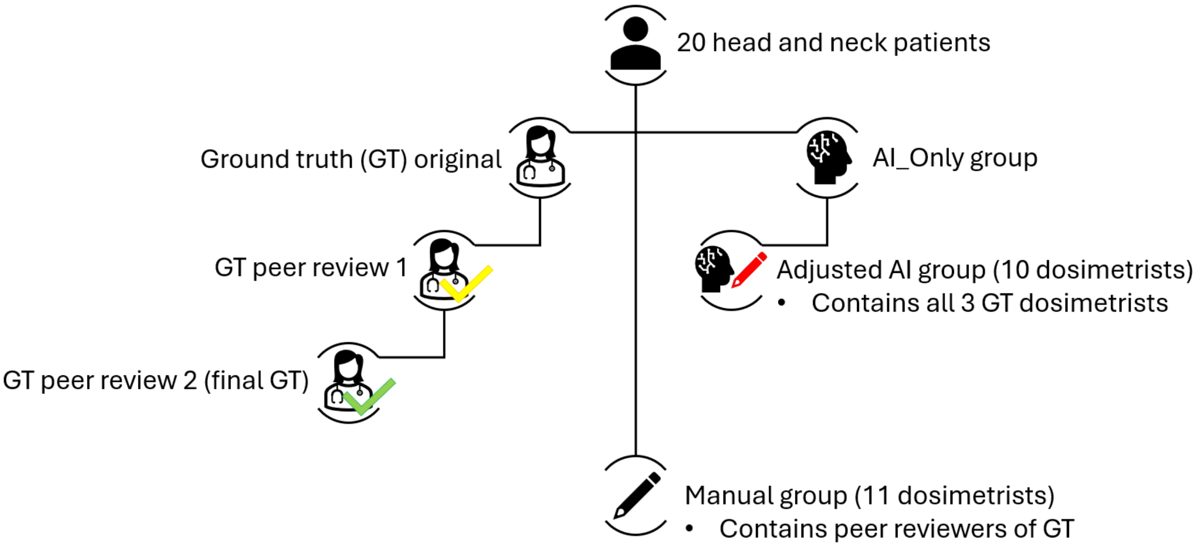

Supplementary Figure 3: A flowchart showing the contouring process in the study. The twenty head and neck patients were contoured manually by a dosimetrist to create the ground truth (GT). The GTs were then peer reviewed by two dosimetrists, each correcting the preceding version. The manual group consisted of eleven dosimetrists (two of which were the peer reviewers of the GT). The GT was established and peer reviewed after the manual group had finished. The patients were sent to the AI software to be contoured, and these AI-contours were then copied and adjusted by ten dosimetrists (including all three of the GT dosimetrists). AI: artificial intelligence.
